# Physical Activity and Health: Does the Pattern Matter?

**DOI:** 10.1093/geroni/igaa057.2861

**Published:** 2020-12-16

**Authors:** Annemarie Koster, Sari Stenholm, Paul Gardiner

## Abstract

It is well-known that physical activity is key in the prevention of many diseases and disability in old age. Much less is, however, known about the pattern of activity in relation to health. While there are differences in how people spread their activity and sedentary behavior over the day or over the week, we don’t know which activity pattern of most beneficial for health. This symposium focuses on patterns of physical activity and sedentary behavior and health in five different studies with accelerometry data in Europe and the USA. Dr. Rosenberg will show how sedentary behavior patterns are associated with various health outcomes in the Adult Changes in Thought (ACT) study. Using data from The Maastricht Study, Dr. Vandercappellen will present how weekly activity patterns, in particular comparing regularly actives to weekend warriors, are associated with arterial stiffness. Dr. Shiroma will show how patterns of physical activity and sedentary behavior, taking the volume, intensity, and frequency of sessions into account, are associated with mortality in the Women’s Health Study. Dr. Caserotti will present the association between physical activity fragmentation and physical function in the SITLESS Study. Dr. Stenholm will present data from the Finish Retirement and Aging Study, using latent class trajectory analyses to identify daily activity patterns and how these patterns are associated with health-related physical fitness. Taken together, this symposium will provide insight into different ways patterns of activity can be operationalized using accelerometer data and if the patterns of activity and sedentary behavior are associated with health.

